# Optimal learning with excitatory and inhibitory synapses

**DOI:** 10.1371/journal.pcbi.1008536

**Published:** 2020-12-28

**Authors:** Alessandro Ingrosso

**Affiliations:** Zuckerman Mind, Brain, Behavior Institute, Columbia University, New York, New York, United States of America; Research Center Jülich, GERMANY

## Abstract

Characterizing the relation between weight structure and input/output statistics is fundamental for understanding the computational capabilities of neural circuits. In this work, I study the problem of storing associations between analog signals in the presence of correlations, using methods from statistical mechanics. I characterize the typical learning performance in terms of the power spectrum of random input and output processes. I show that optimal synaptic weight configurations reach a capacity of 0.5 for any fraction of excitatory to inhibitory weights and have a peculiar synaptic distribution with a finite fraction of silent synapses. I further provide a link between typical learning performance and principal components analysis in single cases. These results may shed light on the synaptic profile of brain circuits, such as cerebellar structures, that are thought to engage in processing time-dependent signals and performing on-line prediction.

## Author summary

A general analysis of learning with biological synaptic constraints in the presence of statistically structured signals is lacking. Here, analytical techniques from statistical mechanics are leveraged to analyze association storage between analog inputs and outputs with excitatory and inhibitory synaptic weights. The linear perceptron performance is characterized and a link is provided between the weight distribution and the correlations of input/output signals. This formalism can be used to predict the typical properties of perceptron solutions for single learning instances in terms of the principal component analysis of input and output data. This study provides a mean-field theory for sign-constrained regression of practical importance in neuroscience as well as in adaptive control applications.

## Introduction

At the most basic level, neuronal circuits are characterized by the subdivision into excitatory and inhibitory populations, a principle called Dale’s law. Even though the precise functional role of Dale’s law has not yet been understood, the importance of synaptic sign constraints is pivotal in constructing biologically plausible models of synaptic plasticity in the brain [1–5]. The properties of synaptic couplings strongly impact the dynamics and response of neural circuits, thus playing a crucial role in shaping their computational capabilities. It has been argued that the statistics of synaptic weights in neural circuits could reflect a principle of optimality for information storage, both at the level of single-neuron weight distributions [6, 7] and inter-cell synaptic correlations [8] (e.g. the overabundance of reciprocal connections). A number of theoretical studies, stemming from the pioneering Gardner approach [9], have investigated the computational capabilities of stylized classification and memorization tasks in both binary [10–13] and analog perceptrons [14, 15], using synthetic data. With some exceptions mentioned in the following, these studies considered random uncorrelated inputs and outputs, a usual approach in statistical learning theory. One interesting theoretical prediction is that non-negativity constraints imply that a finite fraction of synaptic weights are set to zero at critical capacity [6, 15, 16], a feature which is consistent with experimental synaptic weight distributions observed in some brain areas, e.g. input fibers to Purkinje cells in the cerebellum.

The need to understand how the interaction between excitatory and inhibitory synapses mediates plasticity and dynamic homeostasis [17, 18] calls for the study of heterogeneous multi-population feed-forward and recurrent models. A plethora of mechanisms for excitatory-inhibitory (E-I) balance of input currents onto a neuron have been proposed [19, 20]. At the computational level, it has recently been shown that a peculiar scaling of excitation and inhibition with network size, originally introduced to account for the high variability of neural firing activity [21–27], carries the computational advantage of noise robustness and stability of memory states in associative memory networks [13].

Analyzing training and generalization performance in feed-forward and recurrent networks as a function of statistical and geometrical structure of a task remains an open problem both in computational neuroscience and statistical learning theory [28–32]. This calls for statistical models of the low-dimensional structure of data that are at the same time expressive and amenable to mathematical analyses. A few classical studies investigated the effect of “semantic” (among input patterns) and spatial (among neural units) correlations in random classification and memory retrieval [33–35]. The latter are important in the construction of associative memory networks for place cell formation in the hippocampal complex [36].

For reason of mathematical tractability, the vast majority of analytical studies in binary and analog perceptron models focused on the case where both inputs and outputs are independent and identically distributed. In this work, I relax this assumption and study optimal learning of input/output associations with real-world statistics with a linear perceptron having heterogeneous synaptic weights. I introduce a mean-field theory of an analog perceptron in the presence of weight regularization with sign-constraints, considering two different statistical models for input and output correlations. I derive its critical capacity in a random association task and study the statistical properties of the optimal synaptic weight vector across a diverse range of parameters.

This work is organized as follows. In the first section, I introduce the framework and provide the general definitions for the problem. I first consider a model of temporal (or, equivalently, “semantic”) correlations across inputs and output patterns, assuming statistical independence across neurons. I show that optimal solutions are insensitive to the fraction of E and I weights, as long as the external bias is learned. I derive the weight distribution and show that it is characterized by a finite fraction of zero weights also in the general case of E-I constraints and correlated signals. The assumption of independence is subsequently relaxed in order to provide a theory that depends on the spectrum of the sample covariance matrix and the dimensionality of the output signal along the principal components of the input. The implications of these results are discussed in the final section.

## Results

### Mean-field theory with correlations

**Fig 1:**
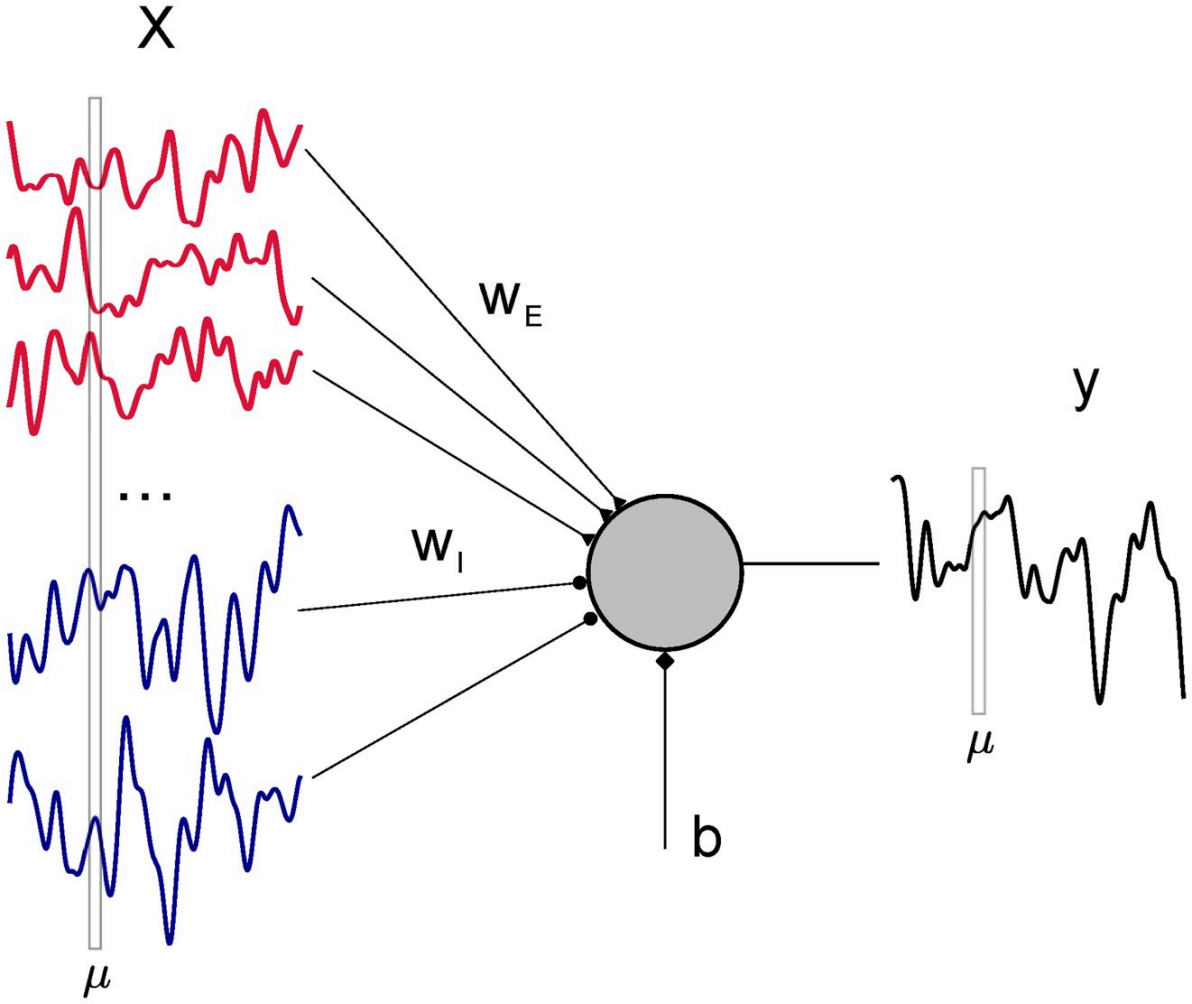
Schematic of the learning problem. A linear perceptron receives *N* correlated signals (input rates of pre-synaptic neurons) *x*_*iμ*_ and maps them to the output *y*_*μ*_ through *N*_E_ = *f*_E_*N* excitatory and *N*_I_ = (1 − *f*_E_)*N* plastic inhibitory weights *w*_*i*_, plus an additional bias current *b*.

Consider the problem of linearly mapping a set of correlated inputs *x*_*iμ*_, with *i* ∈ 1, …, *N* and *μ* = 1, …, *P* from *N*_E_ = *f*_E_*N* excitatory (E) and *N*_I_ = (1 − *f*_E_) inhibitory (I) neurons, onto an output *y*_*μ*_ using a synaptic vector ***w***, in the presence of a learnable constant bias current *b* (Fig 1). To account for different statistical properties of E and I input rates, we write the elements of the input matrix as ${\left(X\right)}_{i\mu }\equiv x_{i\mu }={\bar{x}}_{i}+\sigma_{i}\xi_{i\mu }$ with ${\bar{x}}_{i}={\bar{x}}_{\text{E}}$ for *i* ≤ *f*_E_*N* and ${\bar{x}}_{i}={\bar{x}}_{I}$ for *i* > *f*_E_*N* and the same for *σ*_*i*_. At this stage, the quantities *ξ*_*iμ*_ have unit variance and are uncorrelated across neurons: 〈*ξ*_*iμ*_ *ξ*_*iν*_〉 = *δ*_*ij*_*C*_*μν*_. In the following, we refer to *x* and *y* as signals and *μ* as a time index, although we consider general “semantic”correlations across the patterns ***x***_*μ*_ [34]. The output signal has average $\langle y_{\mu }\rangle =\bar{y}$ and variance $\langle {\left(y_{\mu }-\bar{y}\right)}^{2}\rangle =\sigma_{y}^{2}$. We initially consider output signals *y*_*μ*_ with the same temporal correlations as the input, namely 〈*δy*_*μ*_ *δy*_*ν*_〉 = *C*_*μν*_, where $y_{\mu }=\bar{y}+\sigma_{y}\delta y_{\mu }$.

For a given input-output set, we are faced with the problem of minimizing the following regression loss (energy) function:

$$\begin{array}{c} E(w;\gamma ,x,y)=\frac{1}{2}\sum_{\mu =1}^{P}{\left(\sum_{i=1}^{N}w_{i}x_{i\mu }+b-y_{\mu }\right)}^{2}+\frac{N\gamma }{2}\sum_{i=1}^{N}w_{i}^{2} \end{array}$$ (1)

with *w*_*i*_ > 0 for *i* ≤ *f*_E_*N*, *w*_*i*_ < 0 otherwise. The rationale for using a regularization term lies not only in alleviating ill-conditioning due to input correlations, but also in controlling the metabolic cost of synaptic plasticity and transmission. Preliminary numerical experiments showed that the typical vector ***w*** that solves this sign-constrained least square problem has a squared norm $\sum_{i=1}^{N}w_{i}^{2}=O(1)$, irrespectively of the L2 regularization, as in the special case of i.i.d input/output and non-negative synaptic weights [15]. Synaptic weights *w*_*i*_ are thus of $O(1/\sqrt{N})$, hence the scaling of the regularization term *Nγ* and the bias current $b=I\sqrt{N}$. In order to consider a well defined *N* → ∞ limit for *E* and the spectrum of the matrix *C*, we take *P* = *αN*, with *α* called the *load*, as is costumary in mean-field analysis of perceptron problems [9].

Optimizing with respect to the bias *b* naturally yields solutions ***w*** for which

$$\begin{array}{c} N_{\text{E}}{\bar{w}}_{\text{E}}{\bar{x}}_{\text{E}}+N_{\text{I}}{\bar{w}}_{\text{I}}{\bar{x}}_{\text{I}}+b=\bar{y} \end{array}$$ (2)

where we call ${\bar{w}}_{c}=\frac{1}{N_{c}}\sum_{i\in c}w_{i}=O(1/\sqrt{N})$ the average excitatory and inhibitory weight, with *c* ∈ {E, I}. We call this property *balance*, in that the same scaling is used in balanced state theory of neural circuits [21, 22, 24].

In order to derive a mean-field description for the typical properties of the learned synaptic vector ***w***, we employ a statistical mechanics framework in which the minimizer of *E* is evaluated after averaging across all possible realizations of the input matrix *X* and output *y*. To do so, we compute the free energy density

$$\begin{array}{c} f=-\frac{1}{\beta N}{\left\langle \text{log}\;Z\right\rangle }_{x,y} \end{array}$$ (3)

where *Z* = ∫*dμ* (***w***)*e*^−*βE*^ is the so-called *partition function* and the measure $d\mu (w)=\prod_{i\in \text{E}}\theta (w_{i})dw_{i}\prod_{k\in I}\theta (-w_{k})dw_{k}$ implements the sign-constraints over the synapic weight vector ***w***. The brackets in Eq (3) stand for the quenched average over all the quantities *x*_*iμ*_ and *y*_*μ*_, and the inverse temperature *β* will allow us to select weight configurations ***w*** that minimize the energy *E*. The free energy density *f* acts as a generating function from which all the statistical quantities of interest can be calculated by appropriate differentiation and taking the *β* → ∞ limit. In particular, we will be interested in the (normalized) average loss $\epsilon =\frac{\langle E\rangle }{N}$ and the error $\epsilon_{err}=\frac{1}{2N}\langle {\left|X^{T}w+b-y\right|}^{2}\rangle$, corresponding to the average value of the first term in Eq (1), where ***b*** is a *P*-dimensional vector containing *b* in every element. The average in Eq (3) can be computed in the *N* → ∞ limit with the help of the replica method, an analytical continuation technique that entails the introduction of a number *n* of *formal* replicas of the vector ***w***. A general expression for *f* can be obtained in the large *N* limit using the saddle-point method. The crucial quantity in our derivation is the (replicated) cumulant generating function *Z*_*ξ*,*δy*_ for the (mean-removed) input *x* and output *y*, which can be easily expressed as a function of the eigenvalues λ_*μ*_, *μ* = 1, …, *αN* of the covariance matrix *C*, plus a set of order parameters to be evaluated self-consistently (Methods).

### Critical capacity

The existence of weight vectors ***w***’s with a certain value of the regression loss *E* in the error regime (*ϵ* > 0) is described by the so-called *overlap* order parameter $\Delta {\tilde{q}}_{w}$. In the replica-based derivation of the mean-field theory, overlap parameters are introduced with the purpose of decoupling the *w*_*i*_’s over the *i* index, and represent the scalar-product of two different configurations of the weights ***w*** (Methods: *Replica formalism: ensemble covariance matrix (EC)*). For finite *β*, the quantity $\Delta q_{w}=\beta \Delta {\tilde{q}}_{w}$ represents the variance of the synaptic weights across different solutions. In the asymptotic limit *β* → ∞ of Eq (3), a simple saddle-point equation for $\Delta {\tilde{q}}_{w}$ can be derived when *b* is chosen to minimize Eq (1):

$$\begin{array}{c} \alpha \Delta {\tilde{q}}_{w}{\left\langle \frac{\lambda }{1+\Delta {\tilde{q}}_{w}\lambda }\right\rangle }_{\rho (\lambda )}=\frac{1}{2}-\gamma \Delta {\tilde{q}}_{w} \end{array}$$ (4)

where *ρ*(λ) is the distribution of eigenvalues of *C*.

In the absence of weight regularization (*γ* = 0), we define the critical capacity *α*_*c*_ as the maximal load *α* = *P*/*N* for which the patterns ***x***_*μ*_ can be correctly mapped to their outputs *y*_*μ*_ with zero error. When the synaptic weights are not sign-constrained, the critical capacity is obviously *α*_*c*_ = 1, since the matrix *X* is typically full rank. In the sign-constrained case, *α*_*c*_ is found to be the minimal value of *α* such that Eq (4) is satisfied for $0<\Delta {\tilde{q}}_{w}<+\infty$. Noting that the left-hand side in Eq (4) is a non-decreasing function of $\Delta {\tilde{q}}_{w}$ with an asymptote in *α*, the order parameter $\Delta {\tilde{q}}_{w}$ goes to + ∞ as the critical capacity is approached from the right. We thus find for *γ* = 0 the surpisingly simple result:

$$\begin{array}{c} \alpha_{c}=0.5 \end{array}$$ (5)

**Fig 2:**
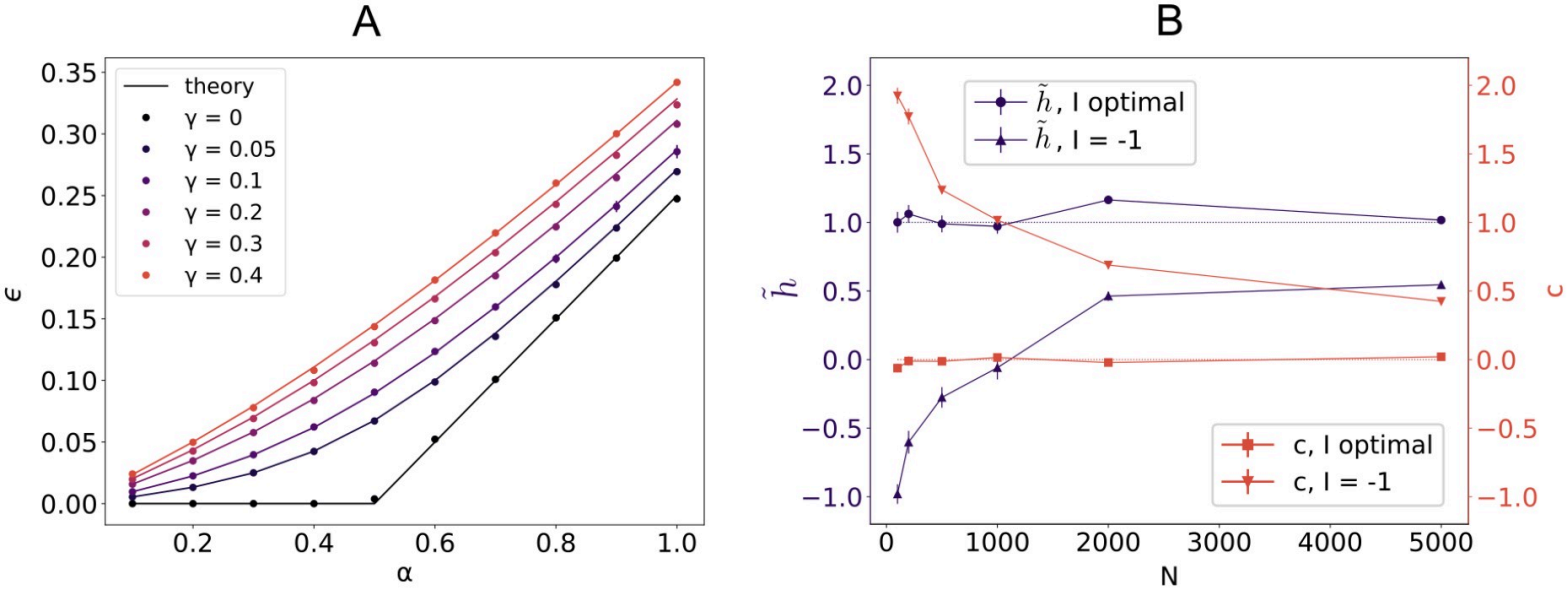
Critical capacity and weight balance. A: Average loss *ϵ* for a linear perceptron with *f*_E_ = 0.8 positive synaptic weights in the case of i.i.d. input *X* and output *y* for increasing values of the regularization *γ*. Parameters: *N* = 1000, ${\bar{x}}_{\text{E}}={\bar{x}}_{I}=\sigma_{\text{E}}=\sigma_{I}=\bar{y}=\sigma_{y}=1$. Each point is an average across 50 samples. Full lines show the theoretical results. B: Mean-field component $\tilde{h}$ (left axis, purple) and weight-input correlation *c* (right axis, red) for increasing dimension *N* in the case where the bias current $b=I\sqrt{N}$ is either learned (*I* optimal) or fixed at the outset (*I* = −1) for *f*_E_ = 1, *γ* = 0.1, *α* = 0.8. Inputs *X* and output *y* are time-correlated with un-normalized Gaussian covariance *C*, *τ* = 10 (see text). The remaining parameters are as in A. The asymptotic value $\tilde{h}=\bar{y}=1$ is highlighted by the purple dotted line, the value *c* = 0 by the red dotted line as guide for the eye.

As shown in Fig 2A in the case of i.i.d. *x* and *y*, the loss has a sharp increase at *α* = 0.5. This holds irrespectively of the structure of the covariance matrix *C* and the ratio of excitatory weights *f*_E_. In Fig 2A, we also show the average minimal loss *ϵ* for increasing values of the regularization parameter *γ*.

In [15], the authors showed that, in the case with excitatory synapses only and uncorrelated inputs and outputs, *α*_*c*_ approaches 0.5 in the limit when the quantity $\frac{\sigma_{y}^{2}{\bar{x}}_{\text{E}}^{2}}{I^{2}\sigma_{\text{E}}^{2}}$ goes to zero, and analyzed which conditions on inputs and outputs statistics lead to maximize capacity. Here we take a complementary approach, where the *x* and *y* statistics are fixed and capacity is optimized within the error regime, so that the optimal bias $I\sqrt{N}$ is well defined in terms of minimizing 〈*E*〉 at any load *α*. The bias optimization leads to a massive simplification of the saddle-point equations and makes results independent of the E/I ratio and the input/output statistics (Methods: *EC, Saddle-point equations*). One may observe that, in the particular case studied by [15], *α*_*c*_ is maximal for very large *I*, due to the divergence of the norm of ***w*** at critical capacity for an optimal bias in the absence of regularization.

The independence of our results with respect to the E/I ratio for an optimal bias current signals a *local gauge invariance*, as observed by [37, 38] for a sign-constrained binary perceptron. Indeed, calling *g*_*i*_ = sign *w*_*i*_, we can write the mean-removed output as $\sum_{i=1}^{N}g_{i}|w_{i}|\sigma_{i}\xi_{i}^{\mu }$ and redefine the *ξ*’s as $g_{i}\xi_{i}^{\mu }$, without changing their occurrence probability. This establishes an equivalence to a linear perceptron with non-negative weights (see [37] for more details), once the mean contribution has been removed. Any residual dependence of *α*_*c*_ or *ϵ* on external parameters must therefore be ascribed to the volume of weights satisfying Eq (2), for a sub-optimal external current *b*.

For a generic value of the bias current *b*, there are strong deviations from the condition in Eq (2). In Fig 2B, we compare the value of the average output $\bar{y}$ with $\tilde{h}\equiv \sum_{c\in \{\text{E},I\}}N_{c}{\bar{w}}_{c}{\bar{x}}_{c}+b$, and also plot the residual term $c=\frac{1}{NP}\sum_{i\mu }\delta w_{i}x_{i\mu }$, where we decomposed the weight vector components as $w_{i}={\bar{w}}_{c}+\delta w_{i}$ for *c* ∈ {E, I}. The quantity *c* measures weight-rate correlations that are responsible for the cancelation of the $O(\sqrt{N})$ bias.

The deviation from Eq (2), shown here for a rapidly decaying covariance of the form $C_{\mu \nu }=e^{-\frac{\left|\mu -\nu \right|}{2\tau^{2}}}$, has been previously described in the context of a target-based learning algorithm used to build E-I-separated rate and spiking models of neural circuits capable of solving input/output tasks [3]. In this approach, a randomly initialized recurrent network *n*_T_ is driven by a low dimensional signal *z*. Its currents are then used as targets to train the synaptic couplings of a second (rate or spiking) network *n*_S_, in such a way that the desired output *z* can later be linearly decoded from the self-sustained activity of *n*_S_. Each neuron of *n*_S_ has to independently learn an input/output mapping from firing rates *x* to currents *y*, using an on-line sign-constrained least square method. In the presence of an L2 regularization and a constant $b\propto \sqrt{N}$ external current, the on-line learning method typically converges onto a solution for the recurrent synaptic weights for which Eq (2) does not hold. As also shown in [3], in the peculiar case of a self-sustained periodic dynamics (in which case off-diagonal terms of the covariance matrix *C*_*μν*_ do not vanish for large *μ* or *ν*) the two contributions $\tilde{h}$ and *c* scale approximately like $\sqrt{N}$ and cancel each other to produce an O(1) total average output $\bar{y}=\tilde{h}+c$. In the effort to build heterogeneous functional network models, the emergence of synaptic connectivity compatible with the balanced scaling thus depends on the statistics of incoming currents. Ad-hoc regularization can be avoided by adjusting external currents onto each neuron.

### Power spectrum and synaptic distribution

**Fig 3:**
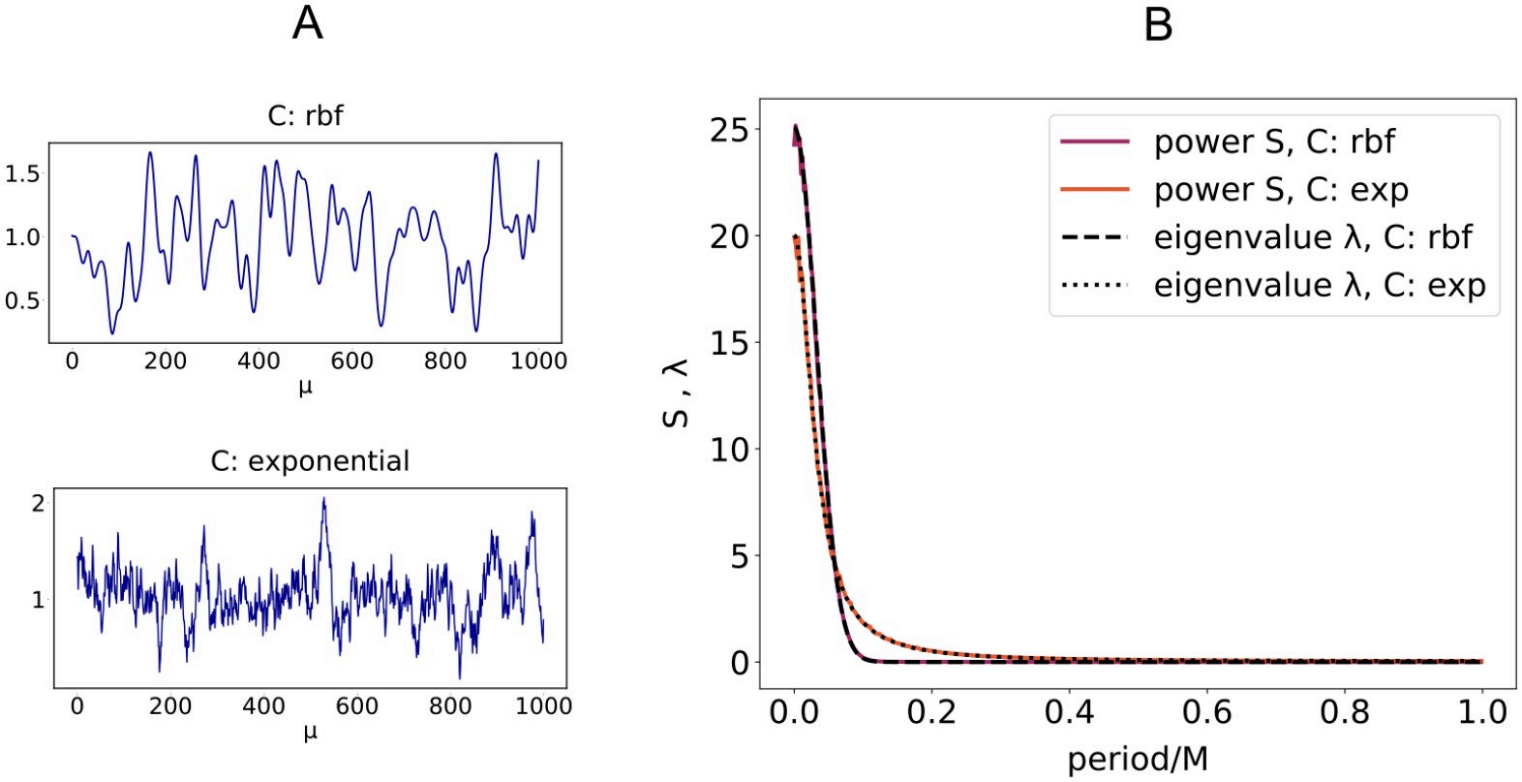
Eigenvalues of *C* and Fourier spectrum. A: Examples of excitatory input signals *x*_*iμ*_ (*i* ∈ E) with two different covariance matrices *C*. Top: rbf covariance, *τ* = 10. Bottom: exponential covariance $C_{\mu \nu }=e^{-\frac{\left|\mu -\nu \right|}{\tau }}$, *τ* = 10. Parameters: ${\bar{x}}_{\text{E}}=1$, *σ*_E_ = 0.3. B: Theoretical eigenvalue spectrum of *C* with *τ* = 10 versus average power spectrum for positive wave numbers across *N* = 2000 independent processes with *P* = 1000 time steps.

The theory developed thus far applies to a generic covariance matrix *C*. To connect the spectral properties of *C* with the signal dynamics, we further assume the *x*_*iμ*_ to be *N* independent stationary discrete-time processes. In this case, *C*_*μν*_ = *C*(*μ* − *ν*) is a matrix of Toeplitz type [39], leading to the following expression for the average minimal loss density in the *N* → ∞ limit:

$$\begin{array}{c} \epsilon =\frac{\sigma_{y}^{2}}{2\pi }\int_{0}^{\pi }d\phi \frac{\lambda (\phi )}{1+\Delta {\tilde{q}}_{w}\lambda (\phi )} \end{array}$$

with $\Delta {\tilde{q}}_{w}$ given by Eq (4). The function λ(*ϕ*) can be computed exactly in some cases (Methods: *Power spectrum and synaptic distribution*) and corresponds to the average power spectrum of the *x* and *y* stochastic processes. Fig 3 shows two representative input signals with Gaussian and exponential covariance matrix *C* (Fig 3A) and a comparison between the average power spectrum of the input and the analytical results for the eigenvalue spectrum of the matrix *C* (Fig 3B). From now on, we use the terms Gaussian or rfb (radial basis function) indistinguishably to denote the un-normalized Gaussian function $C_{\mu \nu }=e^{-\frac{{\left(\mu -\nu \right)}^{2}}{2\tau^{2}}}$.

**Fig 4:**
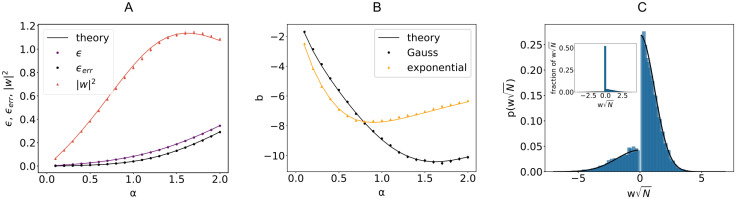
Learning temporally structured signals. A: Minimal loss *ϵ*, error *ϵ*_*err*_ and norm of the weight vector ***w*** as a function of the load *α* for a linear perceptron trained on a time-correlated signal. Covariance matrix *C* is of rbf type with *τ* = 2. Parameters: *N* = 1000, *f*_E_ = 0.8, *γ* = 0.1, ${\bar{x}}_{\text{E}}={\bar{x}}_{I}=\sigma_{\text{E}}=\sigma_{I}=\bar{y}=\sigma_{y}=1$. B: Optimal bias *b* for the two sets of signals with rbf (black curve) and exponential (yellow curve) covariance *C*, with *τ* = 2. Theoretical curves show the value $I\sqrt{N}+\bar{y}$, where *I* has been computed from the saddle-point equations (Methods: *EC, Saddle-point equations*). Parameters as in A. Each point in A and B is an average across 50 samples. C: Probability density of non-zero synaptic weights $w_{i}\sqrt{N}$ of a linear perceptron with *N* = 1000, a fraction *f*_E_ = 0.8 of excitatory weights, trained on *P* = 600 exponentially correlated input *x* and output *y*. The *δ* function in zero is omitted for better visualization. Parameters: *τ* = 10, *γ* = 0.1, ${\bar{x}}_{\text{E}}={\bar{x}}_{I}=1$, *σ*_I_ = 2*σ*_E_ = 0.4. The histogram is an average across 50 realizations of input/output signals. Inset: full histogram of synaptic weights $w_{i}\sqrt{N}$.

As shown in Fig 4A in the case of input *x* and output *y* with rbf covariance, the squared norm of the optimal synaptic vector ***w*** (red curve) is in general a non-monotonic function of *α*, its maximum being attained at bigger values of *α* as the time constant *τ* increases. We also show the minimal loss density *ϵ* and the mean error *ϵ*_*err*_ for *γ* = 0.1. The curves in Fig 4A are the same for any ratio *f*_E_: the use of an optimal bias current *b* cancels any asymmetry between E and I populations. For a finite *γ*, the minimal average loss *ϵ* for a given *f*_E_ decreases as either *σ*_E_ or *σ*_*I*_ increase. For a given set of parameters *f*_E_ and *γ*, the optimal bias *b* will in general depend on the load *α* and the structure of the covariance matrix *C*, as shown in Fig 4B.

Using the same analytical machinery employed for the calculation of the free energy Eq (3), the probability distribution of the typical weight *w*_*i*_ can be easily derived. This can be seen by employing a variant of the replica trick (Methods: *Distribution of synaptic weights*) that links the so-called *entropic part* of *f* to 〈*p*(*w*_*i*_)〉, expressed in terms of the saddle-point values of the same (conjugated) overlap parameters employed thus far. Interestingly, the optimal bias *b* implies that half of the synapses are zero, irrespectively of *f*_E_ and the properties of the covariance matrix *C*. The probability density of the synaptic weights is composed of two truncated Gaussian densities with zero mean for the E and I components, plus a finite fraction *p*_0_ = 0.5 of zero weights.

We show in Fig 4C the shape of the optimal weight distribution for a linear perceptron with 80% excitatory synapses, trained on exponentially correlated *x* and *y* and with a ratio *σ*_I_/*σ*_E_ = 2. It is interesting to note that, in the presence of an optimal external current, both the means of the Gaussian components and the fraction of silent synapses do not depend on the specific properties of input and output signals.

The shape of the synaptic distribution appeared in previous studies both in the binary [8, 11, 13] and linear perceptron [15]. In the linear case with only excitatory synapses [15], for a fixed bias $b=\sqrt{N}$, the fraction of zero E weights is larger than 0.5 at criticality. It generally depends on input parameters and the load in the error region *α* ≤ *α*_*c*_. Let us also mention that a similar property is also apparent in the binary perceptron, where the scale of the typical solutions is set by robustness [13] to input and output noise. For weights $w_{i}=O(1/\sqrt{N})$, the sparsity of critical solutions generically depends on properties of E and I inputs. For weights of O(1/N), robust solutions have a fraction of zero E weights generically larger than 0.5 [6, 11]. When inhibitory synapses are added, their weights are less sparse [11]. Interestingly, in the case without robustness, half of the E and I weights are zero at critical capacity for all *f*_E_ ≥ 0.5.

**Fig 5:**
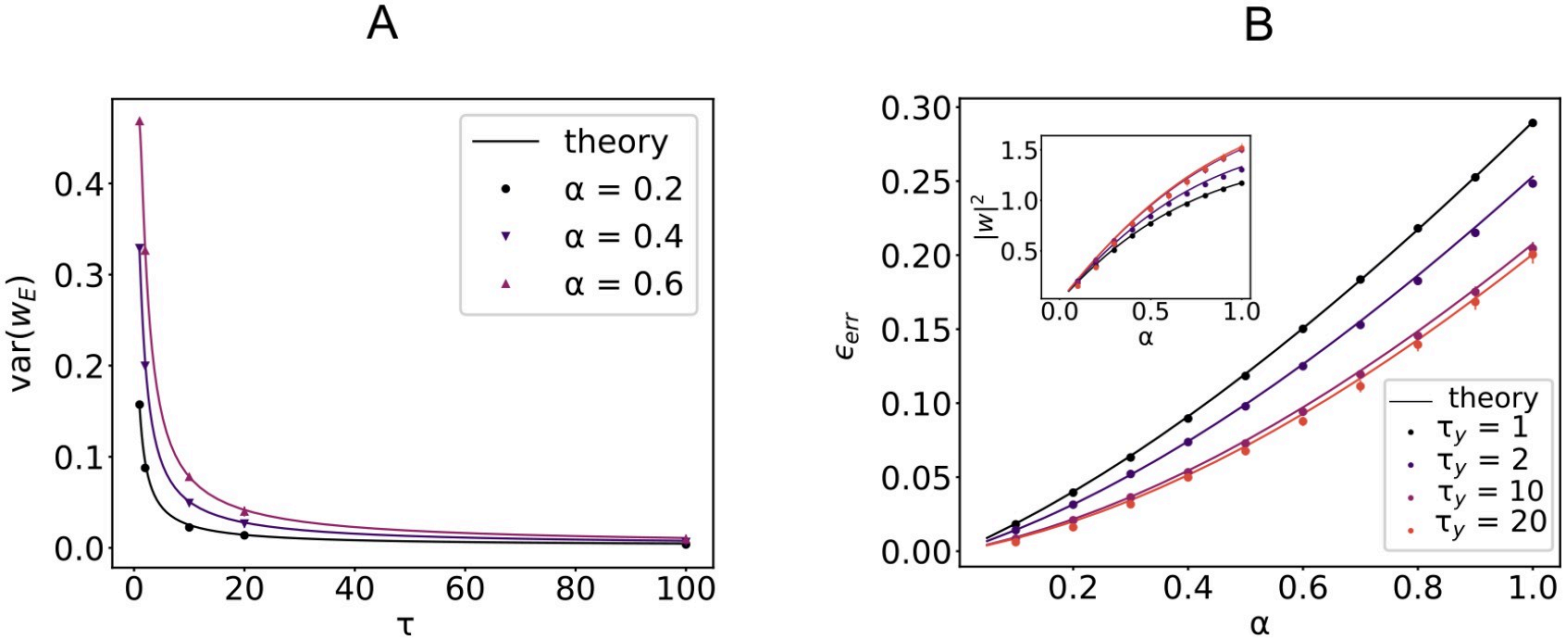
Input/Output time constants and learning performance. A: Variance of synaptic weights (*f*_E_ = 1) for a linear perceptron of dimension *N* = 1000 trained on rbf-correlated signals with increasing time constant *τ* for three different values of the load *α*. Parameters: *γ* = 0.1, ${\bar{x}}_{\text{E}}={\bar{x}}_{I}=\sigma_{\text{E}}=\sigma_{I}=\bar{y}=\sigma_{y}=1$. B: Average error *ϵ*_*err*_ in the case where input and output signals have two different covariance matrices, for increasing time constant *τ*_*y*_ of the output signal *y*. Parameters: *N* = 1000, *f*_E_ = 0.8, *γ* = 0.1, ${\bar{x}}_{\text{E}}={\bar{x}}_{I}=\bar{y}=\sigma_{y}=1$, *σ*_I_ = 2*σ*_E_ = 0.6, *C*^*x*^ rbf with *τ*_*x*_ = 1, *C*^*y*^ rbf with various values of *τ*_*y*_. Inset: norm of the weight vector ***w***. Full lines show analytical results. Points are averages across 50 samples.

The dynamic properties of input/output mappings affect the shape of the weight distribution in a computable manner. As an example, in a linear perceptron with non-negative synapses, the explicit dependence of the variance of the weights on the input and output auto-correlation time constant is shown in Fig 5A for various loads *α*. Previous work considered an analog perceptron with purely excitatory weights as a model for the graded rate response of Purkinje cells in the cerebellum [15]. In the presence of heterogeneity of synaptic properties across cells, a larger variance in their synaptic distribution is expected to be correlated with high frequency temporal fluctuations in input currents. Analogously, the auto-correlation of the typical signals being processed sets the value of the constant external current that a neuron must receive in order to optimize its capacity.

When the input and output have different covariance matrices *C*^*x*^ ≠ *C*^*y*^, a joint diagonalization is not possible in general (Methods: *EC, Energetic part*). We can nevertheless write an expression (Eq (23)) that holds when input and output patterns are defined on a ring (with periodic boundary conditions) and use it as an approximation for the general case. Fig 5B shows good agreement between numerical experiment and theoretical predictions for the error *ϵ*_*err*_ and the squared norm of the synaptic weight vector ***w***, when input and output processes have two different time-constants *τ*_*x*_ and *τ*_*y*_.

### Sample covariance and dimensionality

In the discussion thus far, we assumed independence across the “spatial” index *i* in the input. It is often the case for input signals to be confined to a manifold of dimension smaller than *N*, a feature that can be described by various dimensionality measures, some of which rely on principal component analysis [40, 41]. In order to relax the independence assumption, we build on a framework originally introduced in the theory of spin glasses with orthogonal couplings [42–44] and further developed in the context of adaptive Thouless-Anderson-Palmer (TAP) equations [45–47]. In the TAP formalism, a set of mean-field equations is derived for a given instance of the random couplings (in our case, for a fixed input/output set). In its adaptive generalization [46], the structure of the TAP equations depends on the specific data distribution, in such a way that averaging the equations over the random couplings yields the same results of the replica approach. Here, following previous work in the context of information theory of linear vector channels and binary perceptrons [48–51], we employ an expression for an ensemble of rectangular random matrices and use the replica method to average over the input *X* and output *y*.

Let us write the input matrix ${\left(X\right)}_{i\mu }={\bar{x}}_{i}+\sigma_{i}\xi_{i\mu }$, with *ξ* = *USV*^*T*^, *S* being the matrix of singular values. To analyze the properties of the typical case, we start from a generic singular value distribution *S* and consider i.i.d. output *y*_*μ*_. In calculating the cumulant generating function *Z*_*ξ*,*δy*_, we perform a homogeneous average across the left and right principal components *U* and *V* (Methods: *SC, Energetic part*). Calling *ρ*_*ξξ*^*T*^_(λ) the eigenvalue distribution of the sample covariance matrix *ξξ*^*T*^, we can express *Z*_*ξ*,*δy*_ in terms of a function $G_{\xi ,\delta y}$ of an enlarged set of overlap parameters, which depends on the so-called Shannon transform [52] of *ρ*_*ξξ*^*T*^_(λ), a quantity that measures the capacity of linear vector channels. The resulting self-consistent equations, which describe the statistical properties of the synaptic weights *w*_*i*_, are expressed in terms of the Stieltjes transform of *ρ*_*ξξ*^*T*^_(λ), an important tool in random matrix theory [53] (Methods: *SC, Saddle-point equations*).

**Fig 6:**
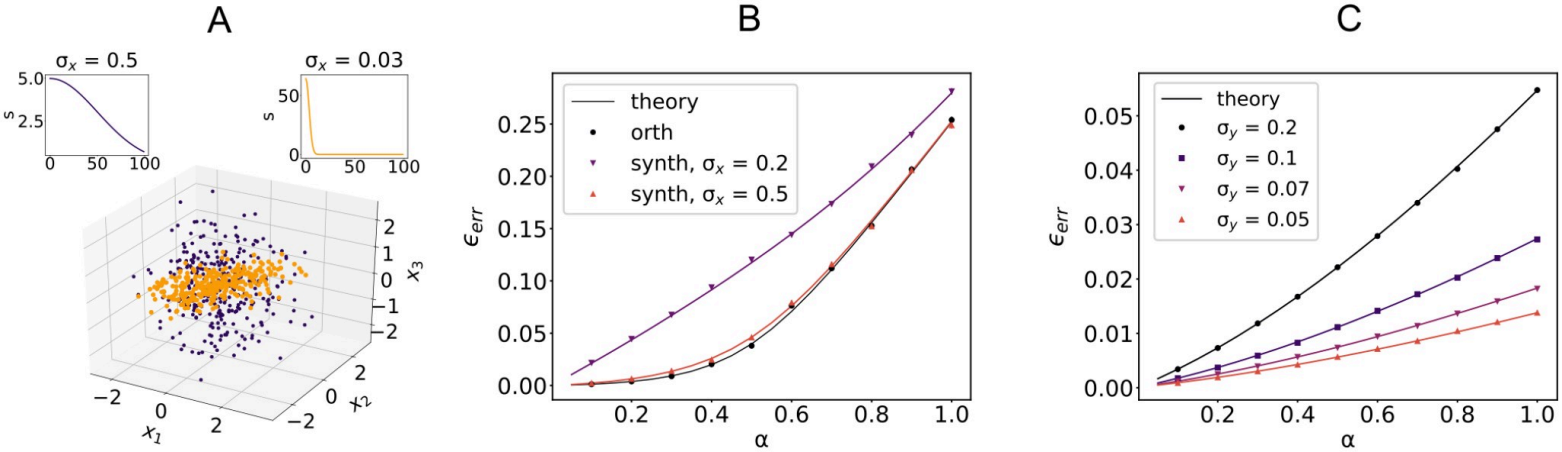
Sample-based PCA and learning performance. A: First three components of inputs ***ξ***_*μ*_ with Gaussian singular value spectrum *s* for two different values of *σ*_*x*_ (color coded top panels). Parameters: *N* = 100, *P* = 300. B: Average error *ϵ*_*err*_ for three different singular value spectra of the input sample covariance matrix: orthogonal model and Gaussian model with increasing *σ*_*x*_ (see main text for definition of *σ*_*x*_). Outputs are i.i.d Gaussian. Parameters: *N* = 1000, *f*_E_ = 0.8, *γ* = 0.1, ${\bar{x}}_{\text{E}}={\bar{x}}_{I}=\bar{y}=\sigma_{y}=1$, *σ*_I_ = 2*σ*_E_ = 0.6. B: Average error *ϵ*_*err*_ for input with orthogonal-type covariance and output *y* with rbf-type covariance with decreasing *σ*_*y*_ (see main text for the definition of *σ*_*y*_). All remaining parameters as in A. Full lines show analytical results. Points are averages across 50 samples.

We show the validity of the mean-field approach by employing two different data models for the input signals. In the first example, valid for *α* ≤ 1, all the *P* vectors ***ξ***_*μ*_ are orthogonal to each other. This yields an eigenvalue distribution of the simple form *ρ*(λ) = *αδ*(λ − 1) + (1 − *α*)*δ*(λ), for which the function $G_{\xi ,\delta y}$ can be computed explicitly [51]. Additionally, we use a synthetic model where we explicitly set the singular value spectrum of *ξ* to be $s(\alpha )=\chi e^{-\frac{\alpha^{2}}{2\sigma_{x}^{2}}}$, with *χ* a normalization factor ensuring matrix *ξ* has unit variance. The shape of the singular value spectrum *s* controls the spread of the data points ***ξ***_*μ*_ in the *N*-dimensional input space, as shown in Fig 6A. As shown in Fig 6B for i.i.d Gaussian output, learning degrades as *σ*_*x*_ decreases, since inputs tend to be confined to a lower dimensional subspace rather than being equally distributed along input dimensions.

For *N* large enough (in practice, for *N* ≳ 500), the statistics of single cases is well captured by the equations for the average case (self-averaging effect). To get a mean-field description for a single case, where a given input matrix *X* is used, we further assume we have access to the linear expansion *c*_*μ*_ of the output *y* in the set {***v***_*μ*_} of the columns of the *V* matrix, namely $y=\bar{y}+\sigma_{y}Vc$. The calculation can be carried out in a similar way and yields, for the average regression loss, the following result:

$$\begin{array}{c} \epsilon =\frac{\alpha }{2}\sigma_{y}^{2}{\tilde{\Lambda }}_{w}{\left\langle \frac{\lambda^{y}}{\lambda^{x}+{\tilde{\Lambda }}_{w}}\right\rangle }_{\lambda^{x},\lambda^{y}} \end{array}$$ (6)

The average in Eq (6) is computed over the eigenvalues λ^*x*^ of the sample covariance matrix, which correspond to the PCA variances, and $\lambda_{\mu }^{y}=c_{\mu }^{2}$ (Methods: *SC, Energetic part*). The quantity ${\tilde{\Lambda }}_{w}$ can be computed from a set of self-consistent equations that link the order parameter $\Delta {\tilde{q}}_{w}$ and the first two moments of the synaptic distribution. To better understand the role of the parameter ${\tilde{\Lambda }}_{w}$, it is instructive to compare Eq (6) with the corresponding result for unconstrained weights, which can be derived from the pseudo-inverse solution w* = (*ξξ*^*T*^ + *γ*)^−1^ *ξy* (Methods: *SC, i.i.d. and unconstrained cases*). The average loss is:

$$\begin{array}{c} \epsilon_{\text{unc}}=\frac{\alpha }{2}\sigma_{y}^{2}\gamma {\left\langle \frac{\lambda^{y}}{\lambda^{x}+\gamma }\right\rangle }_{\lambda^{x},\lambda^{y}} \end{array}$$ (7)

Comparing Eqs (7) and (8), we find that ${\tilde{\Lambda }}_{w}$ acts as an implicit regularization in the sign-constrained case. The mean-field theory is thus carried out through a diagonalization over independent contributions along the components ***v***_*μ*_, with prescribed input and output variances λ^*x*^ and λ^*y*^, respectively. The coupling between different components, induced by the averages 〈⋅〉_*x*,*y*_ and the sign-constraints, is incorporated in the effective regularization ${\tilde{\Lambda }}_{w}$, acting on each component equally, that depends only on the structure of the input *x* (see Eqs (56) and (67) in Methods)).

In Fig 6C, we show results when the dimensionality of the output *y* along the (temporal) components of the input is modulated by taking $c(\alpha )=e^{-\frac{\alpha^{2}}{2\sigma_{y}^{2}}}$. The perceptron performance improves as the output signals spreads out across multiple components ***v***_*μ*_. The case of i.i.d. output is recovered by taking *c*_*μ*_ = 1.

## Discussion

In this work, I investigated the properties of optimal solutions of a linear perceptron with sign-constrained synapses and correlated input/output signals, thus providing a general mean-field theory for constrained regression in the presence of correlations. I treated both the case of known ensemble covariances and the case where the sample covariance is given. The latter approach, built on a rotationally invariant assumption, allowed to link the regression performance to the input and output statistical properties expressed by principal component analysis.

I provided the general expression of the weight distribution for regularized regression and found that half of the weights are set to zero, irrespectively of the fraction of excitatory weights, provided the bias is optimized. The shape of the synaptic distribution has been previously described in the binary perceptron with independent input at critical capacity, as well as in the theory of compressed sensing [54]. I elucidated the role of the optimal bias current and its relation to the optimal capacity and the scaling of the solution weights. This analysis also shed light on the structural properties of synaptic matrices that emerge when target-based methods are used for building biologically plausible functional models of rate and spiking networks.

The theory presented in this work is relevant in the effort of establishing quantitative comparisons between the synaptic profile of neural circuits involved in temporal processing of dynamic signals, such as the cerebellum [55–57], and normative theories that take into account the temporal and geometrical complexity of computational tasks. On the other hand, the construction of progressively more biologically plausible models of neural circuits calls for normative theories of learning in heterogeneous networks, which can be coupled to dynamic mean-field analysis of E-I separated circuits [24, 25, 58].

As shown in this work, the interaction between correlational structure of input signals, synaptic metabolic cost and constant external current shapes the distribution of synaptic weights. In this respect, the results presented here offer a first approximation (static linear input-output associations) to account for heterogeneities of the fraction between E and I inputs to single cells in local circuits. Even though a heterogeneous linear neuron is capable of memorizing *N*/2 associations without error for any E/I ratio, the optimal bias does depend on *f*_E_, its minimal value being attained for *f*_E_ = 0.5. Input current in turn sets the neuron’s operating regime and its input/output properties. Moreover, trading memorization accuracy (small output error *ϵ*_*err*_) for smaller weights (small |***w***|^2^) could be beneficial when synaptic costs are considered (*γ* > 0). It is therefore likely that, for an optimality principle of the 80/20 ratio to emerge from purely representational considerations, dynamical and metabolic effects should be examined all together.

The importance of a theory of constrained regression with realistic input/output statistics goes beyond the realm of neuroscience. Non-negativity is commonly required to provide interpretable results in a wide variety of inference and learning problems. Off-line and on-line least-square estimation methods [59, 60] are also of great practical importance in adaptive control applications, where constraints on the parameter range are usually imposed by physical plausibility.

In this work, I assumed statistical independence between inputs and outputs. For the sake of biological plausibility, it would be interesting to consider more general input-output correlations for regression and binary discrimination tasks. The classical model for such correlations is provided by the so-called teacher-student (TS) approach [61], where the output *y* is generated by a deterministic parameter-dependent transformation of the input *x*, with a structure similar to the trained neural architecture. The problem of input/output correlations is deeply related to the issue of optimal random nonlinear expansion both in statistical learning theory [62, 63] and theoretical neuroscience [41, 64], with a history dating back to the Marr-Albus theory of pattern separation in cerebellum [65]. In a recent work, [28] introduced a promising generalization of TS, in which labels are generated via a low-dimensional latent representation, and it was shown that this model captures the training dynamics in deep networks with real world datasets.

A general analysis that fully takes into account spatio-temporal correlations in network models could shed light on the emergence of specific network motifs during training. In networks with non-linear dynamics, the mathematical treatment quickly gets challenging even for simple learning rules. In recent years, interesting work has been done to clarify the relation between learning and network motifs, using a variety of mean-field approaches. Examples are the study of associative learning in spin models [8] and the analysis of motif dynamics for simple learning rules in spiking networks [66]. Incorporating both the temporal aspects of learning and neural cross-correlations in E-I separated models with realistic input/output structure is an interesting topic for future work.

## Methods

### Replica formalism: Ensemble covariance matrix (EC)

Using the Replica formalism [67], the free energy density is written as:

$$\begin{array}{c} -\beta f=\frac{1}{N}\underset{n\to 0}{\text{lim}}\frac{\partial }{\partial n}\text{log}\;{\left\langle Z^{n}\right\rangle }_{x,y} \end{array}$$ (8)

The function *Z*^*n*^ can be computed by considering a finite number *n* of replicas of the vector ***w*** and subsequently taking a continuation n∈R. The introduction of *n* replicas allows to factorize 〈*Z*^*n*^〉_*x*,*y*_ over individual weights *w*_*i*_, at the cost of coupling different replicas after the averages over the *x* and *y* are performed. Introducing a small set of *overlap order parameters*, factorization across replicas is restored, so that in the large *N* limit the replicated partition function takes the form 〈*Z*^*n*^〉_*x*,*y*_ = *e*^−*βNnf*^. In the following, we will usually drop the subscript in the average 〈⋅〉_*x*,*y*_.

To simplify the formulas, we introduce the O(1) weights $J_{i}=\sigma_{i}\sqrt{N}w_{i}$. In terms of these rescaled variables, the loss function in Eq (1) takes the form:

$$\begin{array}{c} E(w;\gamma ,\xi ,y)=\frac{1}{2}\sum_{\mu =1}^{P}{\left(\sum_{i=1}^{N}\frac{J_{i}}{\sqrt{N}}\xi_{i\mu }+\frac{1}{\sqrt{N}}\sum_{i=1}^{N}\frac{{\bar{x}}_{i}}{\sigma_{i}}J_{i}+I\sqrt{N}-y_{\mu }\right)}^{2}+\frac{\gamma }{2}\sum_{i=1}^{N}\frac{J_{i}^{2}}{\sigma_{i}^{2}} \end{array}$$ (9)

by virtue of $x_{i\mu }={\bar{x}}_{i}+\sigma_{i}\xi_{i\mu }$. We proceed by inserting the definitions $M^{a}=\frac{1}{\sqrt{N}}\sum_{i=1}^{N}\frac{{\bar{x}}_{i}}{\sigma_{i}}J_{ia}+I\sqrt{N}$ and $\Delta_{\mu a}=\sum_{i=1}^{N}\xi_{i\mu }\frac{J_{ia}}{\sqrt{N}}-\sigma_{y}\delta y_{\mu }$ with the aid of appropriate *δ* functions. The averaged replicated partition function 〈*Z*^*n*^〉 is:

$$\begin{array}{cc} \left\langle Z^{n}\right\rangle & =\int \prod_{a}d\mu (J_{a})\int \prod_{\mu a}\frac{d\Delta_{\mu a}du_{\mu a}}{2\pi }\prod_{a}\frac{dM^{a}d{\hat{M}}^{a}}{2\pi /\sqrt{N}}Z_{\xi ,\delta y}\\ & e^{\sum_{a}{\hat{M}}^{a}(\sqrt{N}M^{a}-\sum_{i}\frac{{\bar{x}}_{i}}{\sigma_{i}}J_{ia}-NI)-i\sum_{\mu a}u_{\mu a}\Delta_{\mu a}-\frac{\beta }{2}\sum_{\mu a}{\left(\Delta_{\mu a}+M^{a}-\bar{y}\right)}^{2}-\frac{\beta \gamma }{2}\sum_{ia}\frac{J_{ia}^{2}}{\sigma_{i}^{2}}} \end{array}$$ (10)

where:

$$\begin{array}{c} Z_{\xi ,\delta y}={\left\langle e^{i\sum_{\mu a}u_{\mu a}(\sum_{i}\xi_{i\mu }\frac{J_{ia}}{\sqrt{N}}-\sigma_{y}\delta y_{\mu })}\right\rangle }_{\xi ,\delta y} \end{array}$$ (11)

In Eq 10, we used a Fourier expansion of the *δ* functions and introduced the real variables *u*_*μa*_ as conjugate variables for Δ_*μa*_. Analogously, we employed the purely imaginary ${\hat{M}}^{a}$ for the variables *M*^*a*^. Once the the average is carried out, second cumulants of *ξ* and *δy* get coupled to replica mixing terms of the form *J*_*ia*_ *J*_*ib*_, which can be dealt with by introducing appropriate overlap order parameters $Nq_{w}^{ab}=\sum_{i=1}^{N}J_{ia}J_{ib}$ with the use of *n*(*n* + 1)/2 additional *δ* functions, together with their conjugate variables ${\hat{q}}_{w}^{ab}$. Cumulants of higher order will not contribute to the expression in the large *N* limit. Expanding the *δ* functions for the overlap parameters we get the expression

$$\begin{array}{c} \langle Z^{n}\rangle =\int \prod_{a\leq b}\frac{dq^{ab}d{\hat{q}}^{ab}}{2\pi /N}\prod_{a}\frac{dM^{a}d{\hat{M}}^{a}}{2\pi /\sqrt{N}}e^{\sum_{a}{\hat{M}}^{a}(\sqrt{N}M^{a}-NI)-N\sum_{a\leq b}{\hat{q}}^{ab}q^{ab}+NG_{S}+\alpha NG_{E}} \end{array}$$ (12)

where the two contributions $G_{E}$ and $G_{S}$, respectively called *energetic* and *entropic* part, will be calculated separately in the following for ease of exposition. Owing to the convexity of the regression problem, we use a Replica Symmetry (RS) [67] ansatz $q_{w}^{ab}=q_{w}+\delta_{ab}\Delta q_{w}$ and *M*^*a*^ = *M*.

#### EC, Entropic part

The total volume of configurations ***w***_*a*_ for fixed values of the overlap parameters is given by the *entropic part*:

$$\begin{array}{c} e^{NG_{S}}=\int \prod_{a}d\mu (J_{a})e^{\sum_{a\leq b}{\hat{q}}_{w}^{ab}\sum_{i}J_{ia}J_{ib}-\frac{\beta \gamma }{2}\sum_{ia}\frac{J_{ia}^{2}}{\sigma_{i}^{2}}-\sum_{a}{\hat{M}}^{a}\sum_{i}\eta_{i}J_{ia}} \end{array}$$ (13)

where we called $\eta_{c}=\frac{{\bar{x}}_{c}}{\sigma_{c}}$, with *c* ∈ {E, I}, and *η_i_* = *η*_E_ (*η*_*i*_ = *η*_*I*_) if *i* ∈ E (*i* ∈ I). Using the RS ansatz ${\hat{q}}_{w}^{ab}={\hat{q}}_{w}-\delta_{ab}\frac{{\hat{q}}_{w}+\Delta {\hat{q}}_{w}}{2}$ and ${\hat{M}}^{a}=\hat{M}$, we get:

$$\begin{array}{c} e^{NG_{S}}=\int \prod_{a}d\mu (J_{a})e^{-\frac{1}{2}\sum_{i}(\Delta {\hat{q}}_{w}+\frac{\beta \gamma }{\sigma_{i}^{2}})\sum_{a}J_{ia}^{2}+\frac{{\hat{q}}_{w}}{2}\sum_{i}\sum_{ab}J_{ia}J_{ib}-\hat{M}\sum_{ia}\eta_{i}J_{ia}} \end{array}$$ (14)

Using the explicit definition of the measure $d\mu (J)\propto \prod_{i\in \text{E}}\theta (J_{i})dJ_{i}\prod_{k\in I}\theta (-J_{k})dJ_{k}$, one has, up to constant terms:

$$\begin{array}{c} G_{S}=\sum_{c\in \{\text{E},I\}}f_{c}\text{log}\int_{0}^{\infty }\prod_{a}dJ_{a}e^{-\frac{1}{2}(\Delta {\hat{q}}_{w}+\frac{\beta \gamma }{\sigma_{c}^{2}})\sum_{a}J_{a}^{2}+\frac{{\hat{q}}_{w}}{2}\sum_{ab}J_{a}J_{b}-s_{c}\eta_{c}\hat{M}\sum_{a}J_{a}} \end{array}$$ (15)

where we introduced the notations *f*_I_ = 1 − *f*_E_ and *s*_E_ = −*s*_I_ = 1. In order to disentangle the term ∑_*ab*_ *J*_*a*_*J*_*b*_ = (∑_*a*_ *J*_*a*_)^2^, we employ the so-called *Hubbard-Stratonovich* transformation $e^{\frac{b^{2}}{2}}=\int Dze^{bz}$, where $Dz=dz\frac{e^{-\frac{z^{2}}{2}}}{\sqrt{2\pi }}$. Taking the limit *n* → **0** one gets:

$$\begin{array}{c} G_{S}=\sum_{c\in \{\text{E},I\}}f_{c}\int Dz\;\text{log}\int_{0}^{\infty }dJe^{-\frac{J^{2}}{2}(\Delta {\hat{q}}_{w}+\frac{\beta \gamma }{\sigma_{c}^{2}})+s_{c}(z\sqrt{{\hat{q}}_{w}}-\eta_{c}\hat{M})J} \end{array}$$ (16)

#### EC, Energetic part

In order to compute the *energetic* part, we first need to evaluate the average with respect to *ξ* and *δy* in Eq (11). Performing the two Gaussian integrals we get:

$$\begin{array}{c} Z_{\xi ,\delta y}=e^{-\frac{1}{2}\sum_{\mu \nu }\sum_{ab}q_{w}^{ab}u_{\mu a}u_{\nu b}C_{\mu \nu }^{x}-\frac{\sigma_{y}^{2}}{2}\sum_{\mu \nu }\sum_{ab}u_{\mu a}u_{\nu b}C_{\mu \nu }^{y}} \end{array}$$ (17)

from which:

$$\begin{array}{cc} e^{\alpha NG_{E}} & =\int \prod_{\mu a}\frac{d\Delta_{\mu a}du_{\mu a}}{2\pi }e{}^{-\frac{\beta }{2}\sum_{\mu a}\Delta_{\mu a}^{2}-\frac{1}{2}\sum_{\mu \nu }\sum_{ab}q_{w}^{ab}u_{\mu a}u_{\nu b}C_{\mu \nu }^{x}}\\ & e^{-\frac{\sigma_{y}^{2}}{2}\sum_{\mu \nu }\sum_{ab}u_{\mu a}u_{\nu b}C_{\mu \nu }^{y}+i\sum_{\mu a}u_{\mu a}(M^{a}-\bar{y}-\Delta_{\mu a})} \end{array}$$ (18)

where we performed a translation $\Delta_{\mu a}+M^{a}-\bar{y}\to \Delta_{\mu a}$. In the special case *C*^*x*^ = *C*^*y*^ ≡ *C*, we can use *C* = *V*Λ*V*^*T*^ to jointly rotate **Δ**_*a*_ → *V* **Δ**_*a*_ and ***u***_***a***_ → *V**u***_***a***_, thus leaving scalar products invariant. By doing so, we obtain, within the RS ansatz:

$$\begin{array}{cc} e^{\alpha NG_{E}} & =\int \prod_{\mu a}\frac{d\Delta_{\mu a}du_{\mu a}}{2\pi }e^{-\frac{\beta }{2}\sum_{\mu a}\Delta_{\mu a}^{2}-\frac{1}{2}\sum_{\mu }\sum_{ab}(q_{w}+\delta_{ab}\Delta q_{w})u_{\mu a}u_{\mu b}\lambda_{\mu }}\\ & e^{-\frac{\sigma_{y}^{2}}{2}\sum_{\mu }\sum_{ab}u_{\mu a}u_{\mu b}\lambda_{\mu }+i\sum_{\mu a}\zeta_{\mu }u_{\mu a}(M-\bar{y}-\Delta_{\mu a})} \end{array}$$ (19)

where *ζ*_*μ*_ = ∑_*ν*_ *V*_*μν*_. Using a Hubbard-Stratonovich transformation on the term ∑_*ab*_ *u*_*μa*_ *u*_*μb*_, after some algebra, we obtain:

$$\begin{array}{c} \alpha NG_{E}=-\frac{1}{2}\sum_{\mu }\text{log}(1+\beta \Delta q_{w}\lambda_{\mu })-\frac{\beta }{2}\sum_{\mu }\frac{(q_{w}+\sigma_{y}^{2})\lambda_{\mu }+\zeta_{\mu }^{2}{\left(M-\bar{y}\right)}^{2}}{1+\beta \Delta q_{w}\lambda_{\mu }} \end{array}$$ (20)

Observing that the free energy only depends on *M* through the term ${\left(M-\bar{y}\right)}^{2}$ in $G_{E}$, we conveniently eliminate the quantities *ζ*_*μ*_ at this stage, using the simple saddle-point relation

$$\begin{array}{c} M=\bar{y} \end{array}$$ (21)

thus getting:

$$\begin{array}{c} G_{E}=-\frac{1}{2}{\left\langle \text{log}(1+\beta \Delta q_{w}\lambda )\right\rangle }_{\lambda }-\frac{\beta }{2}(q_{w}+\sigma_{y}^{2}){\left\langle \frac{\lambda }{1+\beta \Delta q_{w}\lambda }\right\rangle }_{\lambda } \end{array}$$ (22)

The brackets 〈⋅〉_λ_ in Eq (22) stand for an average over the eigenvalue distribution *ρ*(λ) of *C* in the *N* → ∞ limit, assuming self-averaging. A similar expression for $G_{E}$ was previously derived in [34] for spherical weights, i.e. $\sum_{i=1}^{N}w_{i}^{2}=1$, in the presence of outputs *y*_*μ*_ generated by a *teacher* linear perceptron. To map Eq (45) in [34] to Eq (22), one substitutes (1 − *q*) → Δ*q*_*w*_ (observing that *q*^*aa*^ = 1 thanks to the spherical constraint) and sets *R* = 0, since the learning task only involves patterns memorization.

When *C*^*x*^ ≠ *C*^*y*^, we can derive a similar expression under the assumption of a ring topology in pattern space (corresponding to periodic boundary conditions in the index *μ*): in this case, both covariance matrices are circulant and may be jointly diagonalized by discrete Fourier transform [33, 34]. In the main text, we show that the expression

$$\begin{array}{c} \alpha G_{E}=-\frac{1}{2N}\sum_{\mu }\text{log}(1+\beta \Delta q_{w}\lambda_{\mu }^{x})-\frac{\beta }{2N}\sum_{\mu }\frac{q_{w}\lambda_{\mu }^{x}+\sigma_{y}^{2}\lambda_{\mu }^{y}}{1+\beta \Delta q_{w}\lambda_{\mu }^{x}} \end{array}$$ (23)

yields good results also when *C*^*x*^ and *C*^*y*^ are covariance matrices of stationary discrete-time processes.

#### EC, Saddle-point equations

All in all, the free energy density in the saddle-point approximation is:

$$\begin{array}{cc} -\beta f & =-\hat{M}I+\frac{\Delta {\hat{q}}_{w}}{2}(\Delta q_{w}+q_{w})-\frac{{\hat{q}}_{w}\Delta q_{w}}{2}\\ & -\frac{1}{2N}\sum_{\mu }\text{log}(1+\beta \Delta q_{w}\lambda_{\mu }^{x})-\frac{\beta }{2N}\sum_{\mu }\frac{q_{w}\lambda_{\mu }^{x}+\sigma_{y}^{2}\lambda_{\mu }^{y}}{1+\beta \Delta q_{w}\lambda_{\mu }^{x}}+\\ & \sum_{c\in \{\text{E},I\}}f_{c}\int Dz\;\text{log}\int_{0}^{\infty }dJe^{-\frac{J^{2}}{2}(\Delta {\hat{q}}_{w}+\frac{\beta \gamma }{\sigma_{c}^{2}})+s_{c}(z\sqrt{{\hat{q}}_{w}}-\eta_{c}\hat{M})J} \end{array}$$ (24)

The saddle-point equations stemming from the entropic part can be written as:

$$\begin{array}{cc} & \Delta q_{w}={\left\langle {\left\langle J^{2}\right\rangle }_{J}\right\rangle }_{z}-{\left\langle {\left\langle J\right\rangle }_{J}^{2}\right\rangle }_{z} \end{array}$$ (25)

$$\begin{array}{cc} & q_{w}={\left\langle {\left\langle J\right\rangle }_{J}^{2}\right\rangle }_{z} \end{array}$$ (26)

$$\begin{array}{cc} & I+\sum_{c\in \{\text{E},I\}}\eta_{c}{\left\langle {\left\langle J\right\rangle }_{J}\right\rangle }_{z}=0 \end{array}$$ (27)

where the averages 〈⋅〉_*J*_ and 〈⋅〉_*z*_ in Eqs (25)–(27) are taken with respect to the mean-field distribution of the *J* weights:

$$\begin{array}{cc} p(J;z) & \propto \sum_{c\in \{\text{E},I\}}f_{c}p_{c}(J;z) \end{array}$$ (28)

$$\begin{array}{cc} p_{c}(J;z) & \propto \theta (s_{c}J)e^{-\frac{J^{2}}{2}(\Delta {\hat{q}}_{w}+\frac{\beta \gamma }{\sigma_{c}})+(z\sqrt{{\hat{q}}_{w}}-\eta_{c}\hat{M})J} \end{array}$$ (29)

where *z* is a standard normal variable and *θ* is the Heaviside function: *θ*(*x*) = 1 when *x* > 0 and 0 otherwise. Eq (25) is obtained by differentiating Eq (24) with respect to ${\hat{q}}_{w}$ and then performing an integration by part in *z*. Eq (26) is easily obtained by subtracting Eq (25) from the saddle-point condition over $\Delta {\hat{q}}_{w}$, while Eq (27) originates from the derivative w.r.t. $\hat{M}$.

In the *β* → ∞ limit, the unicity of solution for *γ* > 0 implies that Δ*q*_*w*_ → 0. We therefore use the following scalings for the order parameters:

$$\begin{array}{cc} & \beta \Delta q_{w}=\Delta {\tilde{q}}_{w} \end{array}$$ (30)

$$\begin{array}{cc} & {\hat{q}}_{w}=\beta^{2}C \end{array}$$ (31)

$$\begin{array}{cc} & \Delta {\hat{q}}_{w}=\beta A \end{array}$$ (32)

$$\begin{array}{cc} & \hat{M}=\beta B\sqrt{C} \end{array}$$ (33)

while $q_{w}=O(1)$. In this scaling, Eqs (25)–(27) take the form:

$$\begin{array}{cc} & \Delta {\tilde{q}}_{w}=\sum_{c\in \{\text{E},\text{I}\}}\frac{f_{c}}{A+\frac{\gamma }{\sigma_{c}^{2}}}H(s_{c}\eta_{c}B) \end{array}$$ (34)

$$\begin{array}{cc} & \frac{q_{w}}{C}=\sum_{c\in \{\text{E},\text{I}\}}\frac{f_{c}}{{\left(A+\frac{\gamma }{\sigma_{c}^{2}}\right)}^{2}}((1+\eta_{c}^{2}B^{2})H(s_{c}\eta_{c}B)-s_{c}\eta_{c}BG(\eta_{c}B)) \end{array}$$ (35)

$$\begin{array}{cc} & \frac{I}{\sqrt{C}}=\sum_{c\in \{\text{E},\text{I}\}}\frac{f_{c}}{A+\frac{\gamma }{\sigma_{c}^{2}}}(\eta_{c}^{2}BH(s_{c}\eta_{c}B)-s_{c}\eta_{c}G(\eta_{c}B)) \end{array}$$ (36)

where $G(x)=\frac{e^{-\frac{x^{2}}{2}}}{\sqrt{2\pi }}$ and $H(x)=\int_{x}^{\infty }Dz$. The two remaining saddle-point equations are:

$$\begin{array}{cc} & C=\frac{1}{N}\sum_{\mu }\lambda_{\mu }^{x}\frac{q_{w}\lambda_{\mu }^{x}+\sigma_{y}^{2}\lambda_{\mu }^{y}}{{\left(1+\Delta {\tilde{q}}_{w}\lambda_{\mu }^{x}\right)}^{2}} \end{array}$$ (37)

$$\begin{array}{cc} & A=\frac{1}{N}\sum_{\mu }\frac{\lambda_{\mu }^{x}}{1+\Delta {\tilde{q}}_{w}\lambda_{\mu }^{x}} \end{array}$$ (38)

Optimizing *f* with respect to the bias $b=I\sqrt{N}$ immediately implies *B* = 0, by virtue of Eq (33), and greatly simplifies the saddle-point equations. Using the scaling assumptions Eqs (30)–(33) together with the saddle-point Eqs (34)–(38), we get Eq (4) in the main text, that is valid for any *α* for *γ* > 0. In the unregularized case (*γ* = 0), it describes solutions in the error regime *α* > *α*_*c*_. The optimal bias *b* can be computed by $I\sqrt{N}$ using Eq (36), that is valid up to an O(1) term equal to $\bar{y}$ (Fig 4B). Keeping only the leading terms in the limit *β* → ∞, Eq (24) can be written as:

$$\begin{array}{cc} -\beta f & =-\beta B\sqrt{C}I+\frac{\beta }{2}Aq_{w}-\frac{\beta C}{2}\Delta {\tilde{q}}_{w}\\ & -\frac{\beta }{2N}\sum_{\mu }\frac{q_{w}\lambda_{\mu }^{x}+\sigma_{y}^{2}\lambda_{\mu }^{y}}{1+\Delta {\tilde{q}}_{w}\lambda_{\mu }^{x}}+\\ & \frac{\beta C}{2}\sum_{c\in \{\text{E},I\}}\frac{f_{c}}{A+\frac{\gamma }{\sigma_{c}^{2}}}((1+\eta_{c}^{2}B^{2})H(s_{c}\eta_{c}B)-s_{c}\eta_{c}BG(\eta_{c}B)) \end{array}$$ (39)

From the definition of the free energy density −*β**Nf*** = 〈log∫*dμ*(*w*)*e*^−*βE*^〉, one has that $\frac{\langle E\rangle }{N}=\partial_{\beta }(\beta f)$. Using Eq (39) and the relevant saddle-point equations, the expression for the average minimal energy density is then:

$$\begin{array}{c} \epsilon =\frac{\sigma_{y}^{2}}{2N}\sum_{\mu }\frac{\lambda_{\mu }^{y}}{1+\Delta {\tilde{q}}_{w}\lambda_{\mu }^{x}} \end{array}$$ (40)

Also, noting that $\partial_{\gamma }E=\frac{N}{2}\sum_{i=1}^{N}w_{i}^{2}$, we can compute the average squared norm of the weights $v=\sum_{i=1}^{N}\langle w_{i}^{2}\rangle$ by *v* = 2∂_*γ*_ *f*. We thus obtain:

$$\begin{array}{c} v=C\sum_{c\in \{\text{E},\text{I}\}}\frac{f_{c}}{\sigma_{c}^{2}{\left(A+\frac{\gamma }{\sigma_{c}^{2}}\right)}^{2}}((1+\eta_{c}^{2}B^{2})H(s_{c}\eta_{c}B)-s_{c}\eta_{c}BG(\eta_{c}B)) \end{array}$$ (41)

The error $\epsilon_{err}=\frac{1}{2N}\langle {\left|X^{T}w+b-y\right|}^{2}\rangle$ can be then computed by $\epsilon_{err}=\epsilon -\frac{\gamma }{2}v$.

### Distribution of synaptic weights

The synaptic weight distribution appearing in Eqs (28) and (29) can be obtained using a variant of the replica trick [6, 67]. Using the expression *Z*^−1^ = lim_*n* → 0_ *Z*^*n*−1^, the density of excitatory weights can be written as:

$$\begin{array}{c} p(w_{\text{E}})=\underset{n\to 0}{\text{lim}}\int \prod_{a}d\mu (w_{a})\delta (w_{11}-w_{\text{E}})e^{-\beta \sum_{a}E(w_{a})} \end{array}$$ (42)

where we picked the first E weight in the first replica *w*_11_ without loss of generality. The calculation proceeds along the same lines as for the entropic part above, since the energetic part does not depend on ***w***_*a*_ explicitely. Isolating the first replica and taking the limit *n* → 0, one gets the expression

$$\begin{array}{c} p(J_{\text{E}})=\theta (J_{\text{E}})\int Dz\frac{e^{-\frac{J_{\text{E}}^{2}}{2}(\Delta {\hat{q}}_{w}+\frac{\beta \gamma }{\sigma_{\text{E}}^{2}})+(z\sqrt{{\hat{q}}_{w}}-\eta_{\text{E}}\hat{M})J_{\text{E}}}}{\int_{0}^{\infty }dJe^{-\frac{J^{2}}{2}(\Delta {\hat{q}}_{w}+\frac{\beta \gamma }{\sigma_{\text{E}}^{2}})+(z\sqrt{{\hat{q}}_{w}}-\eta_{\text{E}}\hat{M})J}} \end{array}$$ (43)

and analogously for the I weights. This expression holds for uncorrelated inputs and outputs and any fixed bias *b*, as well as for any correlated *x* and *y* with optimal bias *b*, where deviations from Eq (2) do not occur. In the *β* → ∞, using the scaling relations Eqs (30)–(33), it can be easily shown that the mean-field weight probability density of the rescaled weights $\sqrt{N}w_{i}$ is a superposition of a *δ* function in zero and two truncated Gaussian densitites:

$$\begin{array}{c} p(\sqrt{N}w)=p_{0}(B)\delta (w)+\sum_{c\in \{\text{E},I\}}f_{c}G(\sqrt{N}w;M_{c},\Sigma_{c})\theta (s_{c}J) \end{array}$$ (44)

where the mean and standard deviation of the Gaussians *G*(⋅;*M*, Σ) are:

$$\begin{array}{cc} & M_{c}=-\frac{\eta_{c}B\sqrt{C}}{\sigma_{c}A+\frac{\gamma }{\sigma_{c}}} \end{array}$$ (45)

$$\begin{array}{cc} & \Sigma_{c}=\frac{\sqrt{C}}{\sigma_{c}A+\frac{\gamma }{\sigma_{c}}} \end{array}$$ (46)

This weight density is valid for *γ* > 0 at any *α* and at critical capacity for *γ* = 0. The fraction of zero weights is given by:

$$\begin{array}{c} p_{0}(B)=f_{\text{E}}H(-\eta_{\text{E}}B)+(1-f_{\text{E}})H(\eta_{I}B) \end{array}$$

### Spectrum of exponential and rbf covariance

For the exponential covariance $C_{\mu \nu }=e^{-\frac{\left|\mu -\nu \right|}{\tau }}$, one has [33]:

$$\begin{array}{c} \lambda (\phi )=\frac{1-x^{2}}{1-2x\;\text{cos}\;\phi +x^{2}} \end{array}$$

with $x=e^{-\frac{1}{\tau }}$. In the rbf case $C_{\mu \nu }=e^{-\frac{{\left|\mu -\nu \right|}^{2}}{2\tau^{2}}}$, the spectrum can be computed by Fourier series [39], yielding

$$\begin{array}{c} \lambda (\phi )=\vartheta_{3}(\frac{\phi }{2},e^{-\frac{1}{2\tau^{2}}}) \end{array}$$

with $\vartheta_{3}(z,q)=1+2\sum_{n=1}^{\infty }q^{n^{2}}\text{cos}(2nz)$ the Jacobi theta function of 3rd type.

### Replica formalism: Sample covariance matrix (SC)

Also in the case of a sample covariance matrix, we are interested in statistically structured inputs and output. An independent average across *x* and *y* would result in a simple dependence on the variance of *y* in the energetic part. To capture the geometric dependence between *x* and *y*, we thus extend the calculations in [50, 51] to the case where the linear expansion of *y*_*μ*_ on the right singular vectors *V*_⋅*μ*_ is known, by taking *δy*_*μ*_ = ∑_*ν*_ *V*_*μν*_ *c*_*ν*_.

In order to compute the replicated cumulant generating function Eq (11), we again introduce overlap parameters $q_{w}^{ab}$, whose volume is given by the previously computed entropic part $G_{S}$. The fact that the entropic part is unchanged in turn implies that the mean-field weight distribution takes the form of Eq (44), with the values of {*A*, *B*, *C*} being determined by a new set of saddle-point equations.

#### SC, Energetic part

Using again the expressions ${\left(X\right)}_{i\mu }={\bar{x}}_{i}+\sigma_{i}\xi_{i\mu }$ and *ξ* = *USV*^*T*^, the replicated cumulant generating function for the joint (mean-removed) input and output is:

$$\begin{array}{cc} Z_{\xi ,\delta y} & ={\left\langle \text{exp}(i\sum_{a}{\tilde{J}}_{a}^{T}S{\tilde{u}}_{a}-i\sigma_{y}c^{T}\sum_{a}{\tilde{u}}_{a})\right\rangle }_{p({\tilde{J}}_{a},{\tilde{u}}_{a})} \end{array}$$ (47)

where we used the change of variables ${\tilde{J}}_{ia}=\sum_{k}U_{ki}J_{ka}$ and ${\tilde{u}}_{\mu a}=\sum_{k}V_{k\mu }u_{ka}$. The average in Eq (47) is taken over the joint distribution $p({\tilde{J}}_{a},{\tilde{u}}_{a})$ resulting from averaging over the Haar measure on the orthogonal matrices *U* and *V*. For a single replica, *Z*_*ξ*,*δy*_ will only depend on the squared norms $Q_{w}=\sum_{i}\frac{{\tilde{J}}_{i}^{2}}{N}$ and $Q_{u}=\sum_{\mu }\frac{{\tilde{u}}_{\mu }^{2}}{P}$ of the two vectors $\tilde{J}$ and $\tilde{u}$. We can therefore write the average in the following way:

$$\begin{array}{c} {\left\langle \text{exp}(i{\tilde{J}}^{T}S\tilde{u}-i\sigma_{y}c^{T}\tilde{u})\right\rangle }_{p(\tilde{J},\tilde{u})}\propto \int \delta ({\left|\tilde{J}\right|}^{2}-NQ_{w})\delta ({\left|\tilde{u}\right|}^{2}-PQ_{u})e^{i{\tilde{J}}^{T}S\tilde{u}-i\sigma_{y}c^{T}\tilde{u}} \end{array}$$ (48)

Introducing Fourier representation for the *δ* functions, we are left with an expression involving an *N* + *P* dimensional Gaussian integral:

$$\begin{array}{cc} & \int \frac{d\Lambda_{w}}{4\pi i}\frac{d\Lambda_{u}}{4\pi i}e^{\frac{N\Lambda_{w}Q_{w}}{2}+\frac{P\Lambda_{u}Q_{u}}{2}}\int d\tilde{J}d\tilde{u}e^{-\frac{\Lambda_{w}}{2}{\left|\tilde{J}\right|}^{2}-\frac{\Lambda_{u}}{2}{\left|\tilde{u}\right|}^{2}+i{\tilde{J}}^{T}S\tilde{u}-i\sigma_{y}c^{T}\tilde{u}}\\ & =\frac{{\left(2\pi \right)}^{\frac{N+p}{2}}}{{\left(4\pi i\right)}^{2}}\int d\Lambda_{w}d\Lambda_{u}e^{\frac{N\Lambda_{w}Q_{w}}{2}+\frac{P\Lambda_{u}Q_{u}}{2}}\text{det}M{}^{-\frac{1}{2}}\text{exp}(-\frac{\sigma_{y}^{2}}{2}(\begin{array}{cc} 0 & c \end{array})M^{-1}(\begin{array}{c} 0\\ c \end{array})) \end{array}$$ (49)

where

$$\begin{array}{c} M=(\begin{array}{cc} \Lambda_{w}1_{N} & -iS\\ -iS^{T} & \Lambda_{u}1_{P} \end{array}) \end{array}$$

and $1_{K}$ is the identity matrix of dimension *K*. Following [51], the determinant can be easily calculated:

$$\begin{array}{cc} \frac{1}{N}\text{log}\;\text{det}M & =\frac{1}{N}\sum_{k=1}^{\text{min}(N,P)}\text{log}(\lambda_{k}^{x}+\Lambda_{w}\Lambda_{u})+\frac{\left(N-\text{min}(N,P)\right)}{N}\text{log}\Lambda_{u}\to \\ & \to {\left\langle \text{log}(\lambda^{x}+\Lambda_{w}\Lambda_{u})\right\rangle }_{\lambda^{x}}+(\alpha -1)\text{log}\Lambda_{u} \end{array}$$ (50)

where the limit is taken for *N* → ∞ and the average is with respect to the eigenvalue distribution *ρ*(λ^*x*^). As for the quadratic portion of the Gaussian integral, calling $\lambda_{k}^{y}=c_{k}^{2}$, we will use the shorthand

$$\begin{array}{c} {\left\langle \frac{\lambda^{y}}{\lambda^{x}+\Lambda_{w}\Lambda_{u}}\right\rangle }_{\lambda^{x},\lambda^{y}}\equiv \frac{1}{P}\sum_{k=1}^{\text{min}(N,P)}\frac{\Lambda_{w}\lambda_{k}^{y}}{\Lambda_{w}\Lambda_{u}+\lambda_{k}}+\frac{1}{P}\sum_{k=\text{min}(N,P)+1}^{P}\frac{\lambda_{k}^{y}}{\Lambda_{u}} \end{array}$$ (51)

Considering now the replicated generating function, all the *n*(*n* + 1) cross-product $J_{a}\cdot J_{b}={\tilde{J}}_{a}\cdot {\tilde{J}}_{b}$ and $u_{a}\cdot u_{b}={\tilde{u}}_{a}\cdot {\tilde{u}}_{b}$ must be conserved via the multiplication of *U* and *V*. Together with the overlap parameters $Nq_{w}^{ab}=\sum_{i}J_{ia}J_{ib}$, we additionally introduce the quantities $Pq_{u}^{ab}=\sum_{\mu }u_{\mu a}u_{\mu b}$, thus obtaining:

$$\begin{array}{c} e^{\alpha NG_{E}}=\int \prod_{\mu a}\frac{d\Delta_{\mu a}du_{\mu a}}{2\pi }Z_{\xi ,\delta y}e^{\sum_{a\leq b}{\hat{q}}_{u}^{ab}(Pq_{u}^{ab}-\sum_{\mu }u_{\mu a}u_{\mu b})-\frac{\beta }{2}\sum_{\mu a}\Delta_{\mu a}^{2}-i\sum_{\mu a}u_{\mu a}(\Delta_{\mu a}-M^{a}+\bar{y})} \end{array}$$ (52)

In the RS case, we again take $q_{w}^{ab}=q_{w}+\delta_{ab}\Delta q_{w}$ and, similarly for the *u*’s, $q_{u}^{ab}=-q_{u}+\delta_{ab}\Delta q_{u}$. In the basis where both $q_{w}^{ab}$ and $q_{u}^{ab}$ are diagonal, the expression for *Z*_*ξ*,*δy*_ becomes

$$\begin{array}{c} Z_{\xi ,\delta y}{=}^{i{\tilde{J}}_{1}^{T}S{\tilde{u}}_{1}-i\sigma_{y}c^{T}\sqrt{n}{\tilde{u}}_{1}}\prod_{b=2}^{n}e^{i{\tilde{J}}_{b}^{T}S{\tilde{u}}_{b}}\rangle \end{array}$$ (53)

so, calling $G_{\xi ,\delta y}=\frac{1}{N}{\text{lim}}_{n\to 0}\text{log}\;Z_{\xi ,\delta y}$, we have:

$$\begin{array}{c} 2G_{\xi ,\delta y}=F(\Delta q_{w},\Delta q_{u})+q_{w}\frac{\partial F(\Delta q_{w},\Delta q_{u})}{\partial \Delta q_{w}}-q_{u}\frac{\partial F(\Delta q_{w},\Delta q_{u})}{\partial \Delta q_{u}}-\alpha \sigma_{y}^{2}K(\Lambda_{w},\Lambda_{u}) \end{array}$$ (54)

with the function *F* given by:

$$\begin{array}{cc} F(x,y) & ={\text{Extr}}_{\Lambda_{w},\Lambda_{u}}\{-{\left\langle \text{log}(\lambda^{x}+\Lambda_{w}\Lambda_{u})\right\rangle }_{\lambda^{x}}-(\alpha -1)\;\text{log}\;\Lambda_{u}+\Lambda_{w}x+\alpha \Lambda_{u}y\}\\ & -\text{log}\;x-\alpha \;\text{log}\;y-(1+\alpha ) \end{array}$$ (55)

and $K(\Lambda_{w},\Lambda_{u})=\Lambda_{w}{\langle \frac{\lambda^{y}}{\lambda^{x}+\Lambda_{w}\Lambda_{u}}\rangle }_{\lambda^{x},\lambda^{y}}$. In Eq (54), it is intended that Λ_*w*_ and Λ_*w*_ are implied by the Legendre Transform conditions:

$$\begin{array}{cc} & \Delta q_{w}=\Lambda_{u}{\left\langle \frac{1}{\lambda^{x}+\Lambda_{w}\Lambda_{u}}\right\rangle }_{\lambda^{x}} \end{array}$$ (56)

$$\begin{array}{cc} & \alpha \Delta q_{u}=\frac{\alpha -1}{\Lambda_{u}}+\Lambda_{w}{\left\langle \frac{1}{\lambda^{x}+\Lambda_{w}\Lambda_{u}}\right\rangle }_{\lambda^{x}} \end{array}$$ (57)

The remaining terms in the energetic part $G_{E}$ involve the $q_{u}^{ab}$ overlaps and their conjugated parameters ${\hat{q}}_{u}^{ab}$. Introducing the RS ansatz ${\hat{q}}_{u}^{ab}={\hat{q}}_{u}+\delta_{ab}\frac{\Delta {\hat{q}}_{u}-{\hat{q}}_{u}}{2}$, the calculation follows along the same lines of the section *SC, Energetic part*. We get:

$$\begin{array}{c} 2G_{E}=\frac{2G_{\xi ,\delta y}}{\alpha }+\Delta {\hat{q}}_{u}(\Delta q_{u}-q_{u})+{\hat{q}}_{u}\Delta q_{u}-\text{log}(1+\beta \Delta {\hat{q}}_{u})-\beta \frac{{\hat{q}}_{u}+{\left(M-\bar{y}\right)}^{2}}{1+\beta \Delta {\hat{q}}_{u}} \end{array}$$ (58)

Eliminating *M*, ${\hat{q}}_{u}$ and $\Delta {\hat{q}}_{u}$ at the saddle-point in Eq (58), $G_{E}$ reduces to:

$$\begin{array}{c} G_{E}=\frac{G_{\xi ,\delta y}}{\alpha }+\frac{q_{u}-\Delta q_{u}}{2\beta }-\frac{q_{u}}{2\Delta q_{u}}+\frac{1}{2}\text{log}\Delta q_{u} \end{array}$$ (59)

#### SC, Saddle-point equations

The final expression for the free energy density

$$\begin{array}{c} -\beta f=-\hat{M}I+\frac{\Delta {\hat{q}}_{w}}{2}(\Delta q_{w}+q_{w})-\frac{{\hat{q}}_{w}\Delta q_{w}}{2}+G_{S}+\alpha G_{E} \end{array}$$ (60)

implies the following saddle-point equations:

$$\begin{array}{cc} & \Delta {\hat{q}}_{w}+\frac{\partial F}{\partial \Delta q_{w}}=0 \end{array}$$ (61)

$$\begin{array}{cc} & \frac{\alpha }{\Delta q_{u}}-\frac{\alpha }{\beta }+\frac{\partial F}{\partial \Delta q_{u}}=0 \end{array}$$ (62)

$$\begin{array}{cc} & {\hat{q}}_{w}=q_{w}\frac{\partial^{2}F}{\partial \Delta q_{w}^{2}}-q_{u}\frac{\partial^{2}F}{\partial \Delta q_{w}\Delta q_{u}}-\alpha \sigma_{y}^{2}\frac{\partial K}{\partial \Delta q_{w}} \end{array}$$ (63)

$$\begin{array}{cc} & \alpha \frac{q_{u}}{\Delta q_{u}^{2}}=q_{u}\frac{\partial^{2}F}{\partial \Delta q_{u}^{2}}-q_{w}\frac{\partial^{2}F}{\partial \Delta q_{w}\Delta q_{u}}+\alpha \sigma_{y}^{2}\frac{\partial K}{\partial \Delta q_{u}} \end{array}$$ (64)

in addition to the entropic saddle-point Eqs (25)–(27), which are unchanged. The saddle-point values of the conjugate Legendre variables Λ_*w*_, Λ_*u*_ greatly simplify the expression for the first and second derivatives of *F*. Indeed, from Eqs (61) and (62) one has:

$$\begin{array}{cc} & \Lambda_{w}=\frac{1}{\Delta q_{w}}-\Delta {\hat{q}}_{w} \end{array}$$ (65)

$$\begin{array}{cc} & \Lambda_{u}=\beta^{-1} \end{array}$$ (66)

or, setting $\Lambda_{w}=\beta {\tilde{\Lambda }}_{w}$:

$$\begin{array}{c} {\tilde{\Lambda }}_{w}=\frac{1}{\Delta {\tilde{q}}_{w}}-A \end{array}$$ (67)

In particular, Eq (56) shows that $\Delta {\tilde{q}}_{w}$ is expressed by a Stieltjes transform of *ρ*(λ^*x*^) and the first term in Eq (55) is its Shannon transform. In the limit *β* → ∞, using the following additional scaling relations for the *u* overlaps:

$$\begin{array}{cc} & q_{u}=\beta^{2}{\tilde{q}}_{u} \end{array}$$ (68)

$$\begin{array}{cc} & \Delta q_{u}=\beta \Delta {\tilde{q}}_{u} \end{array}$$ (69)

we get the expression for the energy density:

$$\begin{array}{c} \epsilon =\frac{\alpha }{2}\sigma_{y}^{2}{\tilde{\Lambda }}_{w}{\left\langle \frac{\lambda^{y}}{\lambda^{x}+{\tilde{\Lambda }}_{w}}\right\rangle }_{\lambda^{x},\lambda^{y}} \end{array}$$

#### SC, i.i.d. and unconstrained cases

Either setting *K* = 0 or λ^*y*^ = 0 reverts back to the i.i.d. output case. In the special case of i.i.d. inputs, the eigenvalue distribution is Marchenko-Pastur

$$\begin{array}{c} \rho (\lambda )=\frac{\sqrt{\left(\lambda -\lambda_{-})(\lambda_{+}-\lambda \right)}}{2\pi \lambda } \end{array}$$ (70)

with $\lambda_{+/-}={\left(1\pm \sqrt{\alpha }\right)}^{2}$, from which $F(\Delta q_{w},\Delta q_{u})=-\frac{\alpha }{2}\Delta q_{w}\Delta q_{u}$. The saddle-point equations are essentially the same as the ones in the section *EC, Saddle-point equations* with $C_{\mu \nu }^{x}=C_{\mu \nu }^{y}=\delta_{\mu \nu }$.

Let us also note that, in the simple unconstrained case, taking for simplicity ${\bar{x}}_{i}=0$ and *b* = 0, the entropic part can be worked out to be, up to constant terms:

$$\begin{array}{c} 2G_{S}=\text{log}\;\Delta q_{w}+\frac{q_{w}}{\Delta q_{w}}-\beta \gamma (\Delta q_{w}+q_{w}) \end{array}$$ (71)

which, at the saddle-point, implies ${\tilde{\Lambda }}_{w}=\gamma$. The mean-field distribution $p(\sqrt{N}w)$ is a zero-mean Gaussian with variance *v* = *q*_*w*_. Using the properties of the Hessian of the Legendre Transform, it is easy to show that:

$$\begin{array}{cc} & q_{w,\text{unc}}=\alpha \frac{\partial K}{\partial \Lambda_{w}}=\alpha {\left\langle \frac{\lambda^{x}\lambda^{y}}{{\left(\lambda^{x}+\gamma \right)}^{2}}\right\rangle }_{\lambda^{x},\lambda^{y}} \end{array}$$ (72)

$$\begin{array}{cc} & \epsilon_{\text{unc}}=\frac{\alpha }{2}\sigma_{y}^{2}\gamma {\left\langle \frac{\lambda^{y}}{\lambda^{x}+\gamma }\right\rangle }_{\lambda^{x},\lambda^{y}} \end{array}$$ (73)

These expressions can also be derived from the pseudo-inverse solution (we take $\bar{y}=0$ for simplicity) w* = (*ξξ*^*T*^ + *γ*)^−1^ *ξy*, by taking an average across *ξ* and *y* in the two expressions:

$$\begin{array}{cc} & v{=}^{*T}w^{*}\rangle =\text{Tr}(\xi yy^{T}\xi^{T}{\left(\xi \xi^{T}+\gamma \right)}^{-2}) \end{array}$$ (74)

$$\begin{array}{cc} & \rangle ={\frac{1}{2}}^{T}y\rangle -\frac{1}{2}\text{Tr}(\xi yy^{T}\xi^{T}{\left(\xi \xi^{T}+\gamma \right)}^{-1}) \end{array}$$ (75)

The i.i.d. output case also follows by performing independent averages over *y* and *ξ*.
